# The ethical challenges raised in the design and conduct of pragmatic trials: an interview study with key stakeholders

**DOI:** 10.1186/s13063-019-3899-x

**Published:** 2019-12-23

**Authors:** Stuart G. Nicholls, Kelly Carroll, Merrick Zwarenstein, Jamie C. Brehaut, Charles Weijer, Spencer P. Hey, Cory E. Goldstein, Ian D. Graham, Jeremy M. Grimshaw, Joanne E. McKenzie, Dean A. Fergusson, Monica Taljaard, on behalf of the Ethics of Pragmatic Trials project

**Affiliations:** 1 Clinical Epidemiology Program-Ottawa Hospital Research Institute (OHRI), Ottawa, ON Canada; 2 Centre for Studies in Family Medicine, Schulich School of Medicine and Dentistry, Western University, London, ON Canada; 3 School of Epidemiology and Public Health, University of Ottawa, Ottawa, Canada; 4 Rotman Institute of Philosophy, Western University, London, ON Canada; 5 Center for Bioethics, Harvard Medical School and Program on Regulation, Therapeutics, and Law at Brigham and Women’s Hospital, Boston, MA USA; 6 Department of Medicine University of Ottawa, Ottawa Hospital Research Institute (OHRI), ON, Ottawa, Canada; 7 School of Public Health and Preventive Medicine, Monash University, Melbourne, Victoria Australia

**Keywords:** Pragmatic trial, Randomized controlled trial, Comparative effectiveness, Research ethics, Informed consent, Benefit-harm analysis, Vulnerable participants, Qualitative, Interviews

## Abstract

**Background:**

There is a concern that the apparent effectiveness of interventions tested in clinical trials may not be an accurate reflection of their actual effectiveness in usual practice. Pragmatic randomized controlled trials (RCTs) are designed with the intent of addressing this discrepancy. While pragmatic RCTs may increase the relevance of research findings to practice they may also raise new ethical concerns (even while reducing others). To explore this question, we interviewed key stakeholders with the aim of identifying potential ethical challenges in the design and conduct of pragmatic RCTs with a view to developing future guidance on these issues.

**Methods:**

Interviews were conducted with clinical investigators, methodologists, patient partners, ethicists, and other knowledge users (e.g., regulators). Interviews covered experiences with pragmatic RCTs, ethical issues relevant to pragmatic RCTs, and perspectives on the appropriate oversight of pragmatic RCTs. Interviews were coded inductively by two coders. Interim and final analyses were presented to the broader team for comment and discussion before the analytic framework was finalized.

**Results:**

We conducted 45 interviews between April and September 2018. Interviewees represented a range of disciplines and jurisdictions as well as varying content expertise. Issues of importance in pragmatic RCTs were (1) identification of relevant risks from trial participation and determination of what constitutes minimal risk; (2) determining when alterations to traditional informed consent approaches are appropriate; (3) the distinction between research, quality improvement, and practice; (4) the potential for broader populations to be affected by the trial and what protections they might be owed; (5) the broader range of trial stakeholders in pragmatic RCTs, and determining their roles and responsibilities; and (6) determining what constitutes “usual care” and implications for trial reporting.

**Conclusions:**

Our findings suggest both the need to discuss familiar ethical topics in new ways and that there are new ethical issues in pragmatic RCTs that need greater attention. Addressing the highlighted issues and developing guidance will require multidisciplinary input, including patient and community members, within a broader and more comprehensive analysis that extends beyond consent and attends to the identified considerations relating to risk and stakeholder roles and responsibilities.

## Introduction

The randomized controlled trial (RCT) is a commonly used experimental research design for generating robust evidence on benefits and harms of health interventions. RCTs are a major research endeavour; a 2010 Institute of Medicine report estimated that there were almost 11,000 ongoing interventional trials, with anticipated enrolment of 2.8 million people [1]. Studies have suggested as many as 75 trials are published on a daily basis [2].

However, RCTs are not homogenous and vary in their intent and design features [3]. Explanatory RCTs aim to generate an understanding of the mechanism of action for the intervention. Accordingly, their design should focus on tightly controlling aspects of delivery and the study environment. Pragmatic RCTs, on the other hand, are intended to have their results directly inform clinical or health policy decisions and should thus mimic as closely as possible the users, settings and circumstances in which it is thought the interventions under evaluation will be used [4].

Much work over the last 10–15 years has sought to articulate the elements of trial design upon which trials may be more explanatory or pragmatic [5–8]. Specifically, work to develop the PRagmatic - Explanatory Continuum Indicator Summary 2 (PRECIS-2) tool identified trials as existing upon a multiaxial continuum, and proposed nine design features upon which trials could be more explanatory or more pragmatic. For example, trial designs that reflect a more pragmatic approach may take place in settings similar to the usual-care settings (as opposed to research facilities), may deploy the intervention using resources or organisational support as would be available in usual care, or may allow for flexibility in the delivery of the intervention at the healthcare professionals’ discretion as may be the case in usual care (see Additional file 1: Table S1 for a full list of the PRECIS-2 domains and descriptions). In addition to study designs that use patient randomization, pragmatic trials may also utilize emerging trial designs. Indeed, in the context of health systems or health policy trials, cluster RCT designs - such as the cluster cross-over design [9], and the stepped-wedge cluster design [9, 10] - are being used to not only evaluate system-level interventions but also individual-level interventions adopted at scale.

Interest in pragmatic RCTs has increased substantially in recent years, most notably since the turn of the century [11, 12]. The increased interest in pragmatic RCTs is likely due to the needs of decision-makers at policy and clinical levels for more rapid, affordable, relevant, applicable research on clinical, policy and service delivery choices, conducted within usual-care health systems, and the needs of research funders to demonstrate the contribution of research tax dollars to health improvements. Further, there is a concern that the apparent effectiveness of interventions tested in explanatory RCTs may not be an accurate reflection of their actual effectiveness in usual practice and, therefore, more pragmatic RCTs are needed to address this discrepancy and improve the ability of decision-makers to successfully select treatment and care options from among competing alternatives [13].

While pragmatic RCTs may increase the relevance of research findings to practice they may also raise new ethical concerns, even while reducing others. Attempts to more closely align research encounters with usual care blur the boundary between research and clinical care, which raises opportunities to streamline consent approaches, but may also generate concerns about understanding. Similarly, attempts to recruit study participants that better reflect the background clinical population may address concerns about the routine exclusion of certain patient groups from clinical trials, while at the same time raising concerns about how patients considered vulnerable ought to be protected [14].

While there is a growing body of empirical research exploring ethical challenges generated by pragmatic RCTs (e.g. [15–20]), few studies draw on actual experiences of investigators, participants, and other stakeholders in the design or conduct of pragmatic RCTs. Moreover, this literature is dominated by studies arising from the USA and may not reflect concerns raised by stakeholders in other jurisdictions where healthcare systems and research regulations differ. Further, this literature has focussed to a great extent on a limited number of topics, such as questions of when written consent approaches may be modified [16, 17, 19, 21], while excluding broader ethical challenges pertaining to other facets of pragmatic RCT designs. There is a need to identify issues drawn from the experiences of teams conducting pragmatic RCTs that reflect their varied attitudes to the underlying concepts of pragmatism, as well as their experiences with widely varied interventions and contexts using different pragmatic RCT designs.

As part of a larger research programme to develop ethical guidance for the design and conduct of pragmatic RCTs [20, 22], we interviewed key stakeholders with the aim of identifying potential ethical challenges that pragmatic RCTs pose.

## Methods

We conducted semi-structured interviews with key stakeholders in the design and conduct of pragmatic RCTs including clinician investigators, methodologists, patient partners within study teams, members of research ethics committees, and knowledge users (e.g. regulators, policy makers). Individuals were eligible for interview if they were involved in the development or implementation of a specific pragmatic RCT, had published works addressing the ethical challenges in pragmatic RCTs, had been engaged in work on the methodological development of pragmatic RCTs, or had been engaged in the governance or oversight of pragmatic RCTs.

### Identification and recruitment of participants

Interviewees were purposively sampled based on their role and jurisdiction (to capture different experiences with trials conducted under different governance structures, such as varying ethics guidelines and regulations). In addition, interviewees were sampled to include those with experience of a range of study designs (such as cluster RCTs or RCTs in which health administrative data are used for outcome ascertainment), and different interventions (including drugs, devices, surgical, and behavioural trials), with the aim of generating a broad range of perspectives on ethical considerations that reflect the heterogeneity in pragmatic RCT designs. Potential interviewees were identified through the study team’s investigator networks, a search of published pragmatic RCTs (including review of two existing reviews of pragmatic RCTs [23, 24]), and searches of research funding programme websites from which self-labelled pragmatic RCTs were identified.

Initial contact and subsequent follow up with potential interviewees were made via email by the study team, except for patient partners or community members of trial teams who were approached through the principal investigator(s) for the identified studies. In this case, the principal or nominated investigator was asked to either provide the contact information of the patient partners or community members involved in their trial, or to forward a study invitation and consent form on behalf of our team and to which patient partners or community members could choose to respond. In all cases, if the identified individual indicated a willingness to participate, a date and time for the interview was arranged. On the agreed date, the consent form was reviewed, and consent obtained to proceed with the interview. Recruitment continued until no new or relevant data were identified, and no new categories were being generated from the data [25].

### Data collection

Interviews were conducted by one researcher (SGN) with training and experience in qualitative methods and research ethics. In addition, a subset of interviews was conducted in tandem with a second member of the research team who also had extensive training in qualitative research approaches (KC). Interviews were conducted in person, by telephone, or by web-conferencing depending on the location and preference of the interviewee. Interview guides were developed and pilot tested with members of the study team. The full guide comprised of three main sections: (1) experiences with pragmatic RCTs; (2) perceptions of ethical issues relevant to pragmatic RCTs (structured around the PRECIS-2 domains [5]); and (3) perspectives on oversight and regulation of pragmatic RCTs (see interview guides in Additional file 1: Material S1 and Additional file 2: Material S2). The study was reviewed and approved by the Ottawa Health Sciences Research Ethics Board (reference 20170435-01H) and all individuals provided informed consent to participate in the study.

Interviews were audio-recorded with consent, and transcribed verbatim by a professional transcription service. De-identified transcripts were made available to interviewees for additional comments, apart from one participant who died between the conduct of the interview and completion of transcription. Additional comments were received from three participants and were incorporated into the final version of the transcript. In one instance a participant did not wish to be audio-recorded and so, with their consent, written notes were taken instead. Finalized versions of transcripts or field notes were imported into qualitative data analysis software (NVivo 11 [26]) for analysis.

### Analysis

Given the present goal of identifying a range of issues, as opposed to developing an underlying theory, the examination of the transcripts was analyzed thematically [27, 28]. Transcripts were coded and labelled inductively, with no prior coding scheme. A strength of the thematic analysis approach is its flexibility insofar as it is theoretically or epistemologically agnostic, allowing it to be used independently of a world view.

Interviews were coded by two researchers (SGN and KC). Each researcher independently coded the same transcripts and met to discuss differences in coding and reach consensus on the major themes. Due to the high degree of consistency in the coding of an initial cohort of texts (*n* = 11), (i.e., the same text segments coded in the same or similar ways) the process was revised such that one researcher (SG) coded the remainder of transcripts, which were then reviewed by the second coder (KC) and discussed to reach consensus. Interim and final analyses were presented to the broader team for discussion before the framework was finalized. The study is reported consistent with the Standards for Reporting Qualitative Research (SRQR) reporting guideline [29].

## Results

Forty-five interviews were conducted between April 2018 and September 2018. Mean interview length was 58 minutes (range 26–103 minutes). Interviewees ranged in terms of their experience; patient partners or community members tended to have been involved in a single trial, while clinical investigators and methodologists had generally been involved in multiple trials. Ethicists and knowledge users, while not directly involved in the design or conduct of trials, often had exposure to multiple trials. Participants also varied in the types of trials to which they had been exposed. These included a range of clinical areas, such as cardiac research, critical care, obstetrics and gynaecology, surgery, and public health. Participants also varied with respect to experiences with different types of trial design, such as cluster RCTs or stepped-wedge designs, and trials that varied with respect to the degree to which they were self-identified as being more or less pragmatic on PRECIS-2 domains. As such, while the interviews used selected examples, comments and overall discussion drew on a breadth of experiences. An overview of participant demographics is presented in Table 1.

**Table 1:** Participant demographics (*N* = 45)

Item	Number	Percent
Country		
USA	16	36%
Canada	10	22%
UK	10	22%
Australia	6	13%
France	1	2%
Other	2	4%
Expertise		
Clinical investigator	10	22%
Methodologist	6	13%
Ethicist or lawyer	7	16%
Knowledge user (e.g., regulator)	6	13%
Patient partner or community member	16	36%
Sex		
Female	22	49%
Experience with research in low-resource settings (*N* = 29)		
Yes	8	28%

In this analysis we focus on the substantive areas where interviewees identified ethical issues that were of particular relevance to pragmatic RCTs (as opposed to issues that were relevant to all RCTs). We identified six major themes with ethical implications: (1) identification of relevant risks from trial participation and determination of what constitutes minimal risk; (2) determining when alterations to traditional informed consent approaches are appropriate; (3) the distinction between research, quality improvement, and practice; (4) the potential for broader populations to be affected by the trial and what protections they might be owed; (5) the broader range of trial stakeholders in pragmatic RCTs, and determining their roles and responsibilities; and (6) determining what constitutes “usual care” and implications for trial reporting. Some specific issues were raised by particular stakeholder groups or in relation to particular aspects of design, and these are reported within the broader themes. We discuss each theme subsequently, and exemplar quotes for all themes are provided in Table 2.

**Table 2:** Exemplar quotes for identified themes

Theme	Example quotes
1. Identification of relevant risks from trial participation and determination of what constitutes minimal risk	1.1 “[A] nother issue would be around things like adverse events. […] [T]hat’s another thing about pragmatic trials, relatively low risk […] but you’re putting it [the intervention] into a high-risk situation. […] [Y] our intervention is actually very low risk, but it’s a question of what do you do then about your adverse events? And I do know examples where — none of the ones I’ve been involved in — but examples where regulators have said you’ve got to collect all your adverse events which could be really, really expensive.” *TE010, Methodologist, UK* 1.2 “I think the, the biggest issue is why something is a risk. I would say it’s a why question. It’s sort of like saying […] both of these are standard treatments, for example, if you’re comparing standard treatments. And if you went to one doctor this would be an accepted treatment. You go to another doctor this is an accepted treatment it’s all kind of haphazard out in the real world anyway so all we’re doing is randomizing so where’s the risks? Right.[…] So it’s, it’s more of a question of conceptualizing how people are affected because of the pragmatic trial #1, and #2 because of that is there any change in their welfare prospect that’s directly attributable to the research? I think that’s the question that’s difficult to answer and not sufficiently explored.” *TE018, Ethicist, USA* 1.3 “And then there’s the issue of should we be going to treat some of them as minimal risk which, at least in the US context, opens up the possibility of doing an expedited review of a clinical trial.” *TE014, Ethicist, USA* 1.4 “So, so here [in Australia] it’s reasonably clear. Here it’s absolute risk, so our system defines risk not in terms of the likelihood or probability of the risk but instead purely in terms of the magnitude.” *TE024, Ethicist, Australia*
2. Determining when alternative consent practices are appropriate	2.1 “[T] he trial that we’re running now, we focused the argument of consent for basically giving consent to follow your data, to have access to your PHI [Personal Health Information] and not actually for the intervention because again, you were going to get one of these two interventions regardless. […] So, we focused our argument on that and we haven’t had any problems with the IRB side of that but what I found interesting was again the consent form’s still 9 pages. […] I find that kind of stuff really takes away from the patient receiving informed consent. They just end up with a huge stack of paper.” *TE005, Clinical investigator, USA* 2.2 “[T] raditional people tend to argue about whether you need to include the detailed long consent in written form versus a more abbreviated consent. The more adventuresome people argue about whether you need consent at all if it’s really answering a question about two modes of practice both of which are used commonly and are in play where there’s no particular reason to pick one over the other.” *TE006, Knowledge user, USA* 2.3 “I think one of the problems that doesn’t afflict investigator-initiated pragmatic studies that does afflict a commercially sponsored trial is the lack of a financial incentive for investigators to recruit patients. … [T] his is where a pragmatic trial is beneficial if you’re doing a large trial with limited data collection, you’re not paying investigators a lot of money so they can generate a huge surplus then that that to me is a good thing.” *TE025, Clinical investigator, Australia* 2.4 “I wonder if that [consent to data, not intervention] might not be the right way to think about this kind of trial [pragmatic trials]. So, it’s not that you’re necessarily consenting to whichever treatment practice is the opted-for treatment practice, I mean if both treatments are standard treatment that might be given in this case. Instead what you’re giving access to is your information and the results of that, how you’re feeling about it and all those kinds of things. I just think that might be a way of thinking about how you could do a consent process for this.” *TE024, Ethicist, Australia* 2.5 “And then there’s the issue of should we be going to treat some of them as minimal risk which, at least in the US context, opens up the possibility of doing an expedited review of a clinical trial which is pretty uncommon otherwise and opens up the possibility of a waiver or modification of informed consent on US regs.” *TE014, Ethicist, USA,* 2.6 “[Y] es, we still quite often face problems with consent. So especially in emergency departments if we’re needing to invoke or trying to invoke emergency consent we still get a lot of kickback about whether we need to go out for a proxy consent at that point in time or whether we must go and find family members who can consent. So there are layers of people who can consent on their behalf. And, you know, very often our argument has been that you need to include these people for whom this is potentially at that stage lifesaving, then we can’t wait. So there is still a lot of discussion around that and a lot of work needs to go into the crafting of the writing of the rationale for why we want to go ahead without consent.” *TE008, Methodologist, UK* 2.7 “And the concerning issues really revolve around the ability to waive consent if the intervention is time critical..” *TE023, Clinical investigator, Australia* 2.8 “I can easily see scandals occurring. But people really have to realize that, if a particular clinic or a research program screws up, they could say wow that was a problem with that researcher and so forth. If you do a pragmatic trial where the entire practice infrastructure of a healthcare system is involved and which endorses what happened [waiver of consent], then from a patient subject’s perspective they’re think this entire healthcare system conspired against me. [laugh] That could happen, you know, if something happened. So I think that I think it’s a big issue.” *TE018, Ethicist, USA* 2.9 “But my worry about all this kind of stuff where you’re doing stuff without getting consent and, and so on is that is that you’ll get these cases where we get used to a practice... Ethics committees become quite comfortable with it. It’s standard practice and then somebody says something to someone and somebody goes hey I’m in a research trial? What? I thought I was getting standard care? And if that’s not handled really, really carefully and it becomes a big media story it hurts recruitment for research. It hurts funding for research and so on.” *TE024, Ethicist, Australia* 2.10 “I think that the, the legal challenges, the ethical challenges, the consent challenges are often under played, you know. It’s recognized but you have the situation where you’ll have researchers having sort of this internal consensus […] and it’s almost like they think that well we [will] resolve this issue without understanding that there’s this legal framework outside, these legal norms outside, that you actually have to satisfy. That researchers or even research policy people can’t do it with a workshop [laugh] and you can’t do it with a consensus meeting. Those things are not going to resolve this issue, because the norms that determine how you’re supposed to proceed are actually designed or actually exist within liberal democracies, […] all it’s going to take is one controversy and the whole thing is gonna unravel. And we saw that with Henrietta Lacks in the United States. We saw that with the Texas blood spot situation where they destroyed was it 6, 7, 8 million samples as a result of a couple of families complaining. So, this matters, right … you can’t forge ahead on wishful thinking and a consensus of researchers. On the contrary you’ve got to resolve it more broadly.” *TE021, Lawyer, Canada*
3. The distinction between research, quality improvement, and practice	3.1 “However, there are huge regulations around anything that you label clinical trial. So, I can go off and behave in the most bizarre fashion as an independent clinician […], I get there will be peer review etc. but basically, I can do what I want. If I want to include people in a clinical trial I have to go through all manner of checks and balances which is fair enough when you’re studying experimental treatments. But when you’re doing more clinical effectiveness trials I think it’s possibly an ethical issue that these things are putting enormous barriers in the way of actually doing the research that needs to be done.” *TE025, Clinical investigator, Australia* 3.2 “[…] there’s this sort of double standard between—people are only interested in research ethics and not practice ethics. And, you know, doctors do unethical things all the time … [S] omeone once said in the New England Medical Journal: if I want to give half my patients a new treatment I have to get ethics committee approval. If I want to give all my patients a new treatment I don’t.” *TE001, Methodologist, UK* 3.3 “I’m quite sceptical about the kind of general case of “oh we need a completely new system”; “oh we need to completely change everything” and so on. I’m more happy with the kind of approach that places like Australia take where they just say it’s all research” *TE024, Ethicist, Australia* 3.4 “[T] he first thing is what kind of ethical framework [should be used with pragmatic trials]? Is it a research ethical framework? Is it a systems or healthcare improvement framework or is it both? […] So that’s for me the biggest question and the most important and the one that this project really needs to sort out clearly.” *TE002, Clinical Investigator, Canada* 3.5 “It’s true of any trial [that discussion happens between patients in and outside the trial] […] so the notion of the kind of often hard line like you can’t talk about this [research outside the trial]; [this distinction], makes no sense on the recruiting side from my perspective. […].” *LM002, Patient partner, USA*
4. The potential for broader populations to be affected by the trial and establishing what protections they might be owed	4.1 “I sometimes get the sense that, if you’re doing a trial on women with post-partum haemorrhage, people think it’s a vulnerable group ─ we’ve got to be extra vigilant, but actually what they do is by making it harder to enrol these women in clinical trials or making it longer (the assessment process), they actually disadvantage them instead of doing anything good for them.” *TE001, Clinical Investigator, UK* 4.2 “[W] here everybody is potentially involved, how do we make sure from a language barrier standpoint or an economic standpoint that people aren’t excluded because they’re not asking the right questions or they don’t understand the material. So I think the medical things [exclusion criteria] made perfect sense to me and I think we dealt with it more on an inclusion side than an exclusion side.” *LM006, Patient partner, USA* 4.3 “The one issue is, how do we trade off, or do we need to trade off, or even, do we need to think about the ethics of those in the trial, those in the control group versus those in the intervention group and those outside the trials who might one day be eligible for these things. […] I think it might be an idea to think about that question against a number of different groups: patients offered the intervention, patients receiving the intervention, patients in the control group, patients who might one day benefit from the information from this trial.” *TE002, Clinical investigator, Canada*
5. The broader range of trial stakeholders in pragmatic RCTs, and determining their roles and responsibilities	5.1 “I guess thinking more expansively, more recently there has been a lot of suggestion or proposals to increase the efficiency of trials by doing registry-based randomized trials, which is again an extension of the same practice [...] And who’s the guardian of that data and who should make these approaches and who should allow potentially a trial to sit overly on that dataset. So guardianship issues, permission issues, consent issues, approach issues, privacy issues.” *TE008, Methodologist, UK* 5.2 “[…] there have been trials that have kind of got both the scientific regulator and the payer’s points built in […] But the objectives I think are quite different. I mean the regulator is looking purely at efficacy and safety so the regulator’s coming to it from the more explanatory end. And the payer is looking at quality of life or qualities or effectiveness. That’s what’s coming into it in the more pragmatic end.” *TE015, Knowledge user, UK* 5.3 “The most interesting IRB ethics thing that I’ve been involved in over the last couple of trials is essentially this off label use […] the FDA or Health Canada they don’t necessarily want to review every single clinical study being done in the country, yet the rules are that if it’s off label they need to. And, at least in the US, the FDA has written very clear guidance documents that basically say the best people to understand whether this off-label use increases the risk to the patients are actually the practitioners and the local IRB. But the local IRBs still don’t want to take on all the liability per se so then they still make you go through the FDA. And that again has been very frustrating because that really is the pragmatic side, right?” *TE005, Clinical investigator, USA* 5.4 “The one thing that I am very clear on and my PPI group were very clear on was that we were not the medical side of the trial. We were the public trial and we took advice from the professors etc. as to what things like the placebo should be.” *LM007, Community member, UK*
6. Determining what constitutes “usual care” and implications for trial reporting	6.1 “[…] obviously it’s one of those things [usual care] where there’s no agreement and I think the most you can do is explain what you mean every time for a given situation. I mean the standard of care is actually more often used in the legal setting in terms of, if you did something wrong. If you’re accused of malpractice, you could say no, this was standard practice. This has been the standard within the community of physicians and for this kind of disorder and so forth. And then there might be instances where an intervention might not be used very often, in fact rarely used for purely practical or accidental historical reasons, but no one would think that that was substandard if you used it, right?” *TE018, Ethicist, USA* 6.2 “usual care is how patients are treated in routine practice if you can profile usually from EHR data sources. So, for example, a patient with diabetes in a given healthcare system, how frequently are they seeing their primary care doctor and their endocrinologist on a yearly basis? What proportion of them are on insulin or other diabetes agents? And how does that conform to recommended practice guidelines? So guidelines define some of that but your local care pattern is going to differ a little bit from the guideline. But you can define that and it will differ by region and environment.” *TE007, Clinical investigator, USA* 6.3 “I think that [usual care] fits in nicely with the whole pragmatic/explanatory thing. So on the pragmatic side my interpretation would be usual care is at the individual practitioner level […]And on the explanatory side usual care will be some national, regional standard that everybody can agree on and then therefore you can standardize usual care to sort of a standard of care.” *TE005, Clinical investigator, USA* 6.4 “Well usual care’s not a single thing. Usual care’s whatever that patient would have got in contact with whatever clinician they happened to be in contact with in whatever clinic they happened to be in. And so, the problem with usual care which explanatory trialists really struggle with and, and so they should and so should we as more pragmatic trialists, is the care that the control group gets in a usual care trial is going to be pretty varied” *TE002, Clinical Investigator, Canada* 6.5 “I think others have argued well once you get consent from someone you’re obligated in the usual care arm to give them the standard of care which is almost always higher than the usual care that people actually get. I don’t subscribe to that, but I also think there are many trials in which to answer the scientific question and, and the pragmatic question you actually do need to give standard of care.” *TE006, Knowledge user, USA* 6.6 “[…] are you compromising standard of care by having them [specific patient groups] in the study? Meaning that if you withhold standard of care therapies or treatments as part of a patient’s participation in the study, that’s not ethical. The study should actually look at things on top of that or in comparison to it, but withholding it is not proper.” *TE007, Clinical investigator, USA* 6.7 “the major concern, of course, is that routine practice is changing all the time. Well we hope it is, I mean, there is all this money going in for infrastructure and for […] capacity building on a routine basis. It’s much harder, then, to think of a pragmatic trial that will exactly mirror routine practice that is itself flexible. So rather than the intervention being flexible it’s […] whether the intervention evaluated in a pragmatic trial is […] going to map on to the intervention as it appears in routine practice [and] to how relevant the data will be.” *TE020, Ethicist, UK* 6.8 “The other part of pragmatic trial is that the intervention could be less described or some part of the intervention could be different from one physician to another in pragmatic trials. You could either explain very well all the different components of your intervention or you could decide in your pragmatic trials that some of the components of the interventions are to the discretion of the physician […] Even if it is stuff like that it must be at least reported in the paper and it’s not always reported.” *TE016, Clinical Investigator, France*

*RCT* randomized controlled trial

### Identification of relevant risks from trial participation and determination of what constitutes minimal risk

Risk was a recurring theme. It was raised in relation to the types of risks that should be deemed relevant (for example, in determining eligibility criteria or for disclosure in consent procedures) and how these differed from usual clinical care, how risks should be incorporated and traded off within benefit-harm analyses, when studies should be considered to meet the designation of “minimal risk”, and implications of risk designation on the regulations that need to be followed.

With respect to eligibility, interviewees discussed the risks to participants and which risks should be considered relevant to setting eligibility criteria. Interviewees commented on the benefits of pragmatic RCTS and drew distinctions between those patients who may be at higher risk of adverse outcomes *irrespective* of the intervention at hand and those that may be at a higher risk *because of* the intervention (see Quote 1.1). This was noted due to the perception that pragmatic trials of minimal risk interventions can be employed in populations that are of poor health. This was particularly relevant in relation to broader considerations of potential benefits and harms to participants in a trial, and how this was managed within governance processes such as ethics review or other regulatory reviews. For example, interviewees raised the point that risk assessments may be more complicated in pragmatic RCTs with a more heterogenous population in which risks may accrue differently to different sub-populations. Specific reference was also made to the risks of trial interventions or comparators when these were claimed to be usual care or standard care. Some participants suggested that when the intervention(s) or comparator(s) was described as usual care, it should be considered to be low or minimal risk.

Others suggested that head-to-head trials of two usual care interventions did not necessarily mean that there was no risk to trial participation and rather it depended on whether participating created a change in the participants welfare (Quote 1.2). This fed back into broader questions of what should be considered a risk and the comfort with conducting RCTs in contexts where patients had a poor prognosis.

A key aspect to the discussion of risk was how risks were managed within the oversight of pragmatic RCTs, such as ethics review processes or regulatory requirements. An aspect of the discussion relating to risks of usual care interventions was whether this opened opportunities for different types of review process (see Quote 1.3). On this point, it was noted that different jurisdictions may apply different standards to determine the review requirements with some jurisdictions applying assessments of relative risk of participation in the RCT (i.e., additional risks to participants generated by the research study) while others may employ assessments of absolute risk (i.e., risk of an outcome that is based purely on the likelihood of the outcome irrespective of that incremental differences between research and care), which may lead to different process requirements (Quote 1.4). The importance of risk determinations, and in particular, a determination that a trial poses minimal risk to participants, for governance decisions was emphasised. It was noted that, in some cases, a designation of minimal risk could serve as a necessary condition for expedited or delegated review of research protocols, or waiver of consent. However, this again was subject to jurisdictional variation with respect to the types of studies that could be considered under an expedited review process, even if deemed to be minimal risk.

### Determining when alternative consent practices are appropriate

Interviewees discussed a variety of alternative consent processes, ranging from verbal discussion and acknowledgement, to deferred consent, proxy consent, opt out approaches (with and without notification), and waiver of consent. While interviewees raised many concerns that are already well-documented within the broader clinical trials literature (such as perceptions that ethics review committees spend substantial time reviewing and commenting on consent forms at the expense of consideration for other aspects of study design), three particular consent-related issues were emphasized with respect to pragmatic RCTs, namely: (1) the potential for consent to be simplified or modified from standard written consent practices and the circumstances where this may be legitimate; (2) the separation of consent to trial interventions and consent to data collection, and; (3) the acceptable instances where consent can be waived completely.

There was much discussion of (and consternation at) existing consent approaches, and a desire to simplify these (Quotes 2.1–2.2). For example, an integrated consent approach in which clinicians approach participants for consent to the RCT within the clinical encounter was discussed as an alternate approach. While recruitment of trial participants by their treating clinician is not a unique issue in pragmatic RCTs, it was flagged as potentially being more pronounced in pragmatic RCTs due to the closer integration of research and clinical care. This was raised as a consideration due to the perception that, as several interviewees commented, pragmatic RCTs were less likely to have commercial investment and so may have lower budgets that limit infrastructure to support recruitment. While some interviewees raised concerns about the potential for patients to feel a pressure to participate when recruited by their treating physician, some saw the lack of financial support for recruitment as ethically advantageous. This was based on the perception that the limited resources prevent physicians from being remunerated based on the number of people they recruit, thus removing the potential financial incentive to over recruit or recruit marginally eligible or unsuitable participants (Quote 2.3).

While alternative approaches to traditional written informed consent were discussed, participants varied in their enthusiasm for adapting or altering consent approaches. Rather than seeking alternate approaches that sought to capture a single consent for all aspects of the study (i.e. that covered enrollment, interventions, and data collection), some argued it may be appropriate to consider to what the trial participants should consent. For example, one interviewee provided an analogy to education research arguing that when an intervention is consistent with accepted standards and is unavoidable, then it may be more appropriate to disentangle consent to the intervention and consent to data collection (Quote 2.4). In this case, consent would be sought only for data collection in the study, as opposed to the exposure to the intervention that could not be avoided without extensive means.

A particular aspect of discussion was the circumstances under which one could consider a waiver of consent, thus avoiding consent entirely. When waiver of consent was raised, participants discussed the criterion of impracticability, as well as the aforementioned criterion of minimal risk (Quote 2.5), and under what circumstances these could arise. When considering impracticability, discussion revolved around the urgency of the intervention, the population being studied, resources required (both financial and human), or setting-related characteristics that may make it impracticable to get individual consent (Quotes 2.6–2.7).

A note of caution, however, was raised on the use of waivers of consent. Several respondents emphasized the potential fall-out of failing to manage the use of waivers appropriately. Particular caution, almost exclusively raised by the ethicist and legal stakeholder participants, referred to the need for consent approaches to be consistent with legal norms and requirements, but also how negative media coverage of studies using a waiver of consent may serve to erode public trust and have a harmful impact on research through reduced recruitment or funding (Quotes 2.8–2.10). In making these points, respondents described historic research ethics cases (such as organ retention at Alder Hey Children’s Hospital in the UK and at Greenlane Hospital in New Zealand [30, 31]).

### The distinction between research, quality improvement, and practice

Frustration was expressed at the lack of agreed criteria for demarcating clinical practice, quality improvement, and research. While the lack of clarity regarding the distinction was commented on by all stakeholder groups, the context in which the comments arose differed between the patient partners and community members, and other groups such as the clinical investigators and methodologists. The clinical investigators and methodologists tended to discuss the distinction in the context of the research ethics and regulatory requirements (Quotes 3.1 and 3.2); with the perception that the distinction between research and practice was ill-defined, and that criteria such as intent to publish were inappropriate. Other interviewees argued that when the interventions under investigation are used in routine clinical practice, there may be negligible difference between the research and practice; referred to by one participant as patients receiving “randomized” care rather than “random” (i.e., arbitrary) care. Thus, a common claim was that flawed criteria were being used to make the distinction between activities that needed regulatory oversight and those that did not and that this was resulting in an unnecessary administrative burden for research when in fact the patient’s situation was perceived to be no different than what it would be in usual care, without the research. Others suggested that there may be some criteria—such as generalizability of the results—that may be relevant to distinguish research from practice (Quote 3.3). However, perceptions of appropriate regulatory perspectives varied, with some respondents arguing for the development of new health systems ethics frameworks, while others argued that existing frameworks were likely sufficient, but greater exposure to pragmatic RCTs is needed (Quote 3.4).

By contrast, patient partners and community members reflected on their real-life experiences of patient care and participation in research, and how the boundaries between research and practice were blurred or non-existent for them. Specifically, they noted how patients transition between research and care with an information flow between the two or how information about trials is exchanged informally between patients in their clinical care (Quote 3.5). As such, interviewees problematized the idea of a clear line between the research and clinical aspects of care.

### The potential for broader populations to be affected by the trial and establishing what protections they might be owed

Interviewees discussed how pragmatic RCTs may include a broader range of patients than explanatory trials that might only have included a subset of a clinical population, and that this raised challenges of identifying the extent to which particular groups or individuals may be affected by the trial and the protections owed to them. Others discussed how pragmatic RCTs of health systems or health policy trials may have an impact on individuals not traditionally considered to be research participants and raised questions on how responsible parties should respond. This was not just in relation to who may be affected in a material sense, but also those who may expect to have legitimate claims of those conducting the trial.

One particular area of concern was equity and justice in relation to the participants who were recruited within trials. Interviewees raised concerns about groups, such as pregnant women, children, and those patients with co-morbidities, being excluded from explanatory trials and how pragmatic RCTs may be beneficial in this regard because they were more inclusive of the range of patients who would be seen in usual practice (Quote 4.1). Several patient partners and community members also pointed out the potential for inequalities within trials and how there may be systemic barriers to participation, such as limited literacy levels or home commitments, even when participants met inclusion criteria (Quote 4.2). The exclusion of participants based on elements not associated with eligibility criteria was viewed as a potential threat for pragmatic RCTs when compared to explanatory RCTs. However, it was also noted that in some jurisdictions, such as France, certain patient characteristics, such as ethnicity, cannot be collected, which may preclude consideration of certain equity issues within the trial.

Interviewees also reflected on whether and to what extent pragmatic RCTs should have a broader consideration with respect to the range of individuals or groups affected by the trial, in terms of those affected during the conduct of the trial, but also subsequently affected by the results. One example given was how members of the public who provide cardiopulmonary resuscitation may be affected by research on out-of-hospital resuscitation but have limited contact with a study. In this instance, the interviewee raised the question of what those members of the public were owed in terms of research protections or follow up. Other examples included whether the impact of an intervention on family members who may support frail or cognitively impaired participants should be captured within studies. Others raised the question of whether there is a need to consider ethical obligations to the background populations from which trial participants are drawn in pragmatic RCTs or even to future generations of patients. The question was then raised as to what rights or protections these groups are owed (Quote 4.3). Similarly, the question arose as to how we should consider stakeholders groups who occupy multiple roles. For example, healthcare staff may be the target of an intervention while simultaneously having to collect data from patients and families or provide feedback on an intervention and so may be considered participants, but also part of the trial intervention. As such, issues were threefold: which individuals fall within the bounds of those owed protections within the context of the trial? On what basis are these boundaries drawn? And what are the identified individuals or groups owed in terms of protections or rights relative to the trial; that is, can we establish differential protections or responses to these individuals depending on the extent to which they are affected by the trial?

### The broader range of trial stakeholders in pragmatic RCTs, and determining their roles and responsibilities

Interviewees noted that pragmatic RCTs employ a range of designs or design features, such as comparative effectiveness research on interventions routinely offered in usual-care settings, and may evaluate a wider range of interventions, such as policies, than traditional explanatory trials, which often focus on new drug treatments or technologies. Because of this broad range of designs, contexts, and interventions—including complex interventions—participants indicated that pragmatic RCTs potentially engage a broader range of stakeholders in their design or conduct.

While interviewees identified commonly cited groups, such as trial steering committees, data safety and monitoring boards, clinicians, and researchers as stakeholders with important roles and responsibilities, they also identified other groups. These included healthcare administrators, health maintenance organizations, clinical research regulators (such as Health Canada or the Food and Drug Administration), and stakeholder advisory committees that include a broader range of perspectives such as patient partners. This broader range of stakeholders potentially engaged within a trial predicated further ethical considerations in terms of not only negotiating who had ethical responsibilities as part of the trial, but also how the identified responsibilities were divided between the different stakeholders. For example, when a trial accesses registry or health administrative data, how does one determine who the relevant stakeholders are and what they are responsible for? (Quote 5.1).

As an example, it was observed that some trials may attempt to satisfy multiple stakeholders (such as payers and regulators) in an effort to be more efficient, yet the differing perspectives and needs of these stakeholders also had the potential to raise tensions within the trial, for example in terms of the design choices that may better satisfy one stakeholder than another (Quote 5.2). One participant noted how a trial in which regulated drugs were used “off label” led to the regulator and research ethics committee trying to ascribe responsibility to the other, suggesting that the division of responsibilities between multiple stakeholders may be contested (Quote 5.3). Others noted how within a trial team there would be areas for each stakeholder that were deemed to be legitimately within their scope for comment and others that were outside their scope. For example, several patient partners and community members made reference to identifying and raising issues of inequality within the trial as part of their perceived role within the trial team, but indicated methodological aspects of study design may be outside their remit or expertise (Quote 5.4).

As such, interviewees raised the identification of stakeholders who have roles to play in the design and conduct of pragmatic RCTs as a necessary prerequisite to then determining what responsibilities, including their duties to trial participants, those stakeholders have, but that these stakeholders may extend beyond clinical investigators and their teams.

### Determining what constitutes “usual care” and implications for trial reporting

Interviewees demonstrated varied interpretations of the term “usual care” sometimes using the term interchangeably with “routine care” or with “standard of care”. Some pointed to specific differences in terminology. For example, in a medical-legal context, the term “standard practice” may be used to describe acceptable practice as opposed to a more epidemiological use where “usual care” tended to be used to describe the care most frequently used for a particular clinical condition (see Quote 6.1).

As previously mentioned, interviewees had different views about how usual care should be defined, which influenced perceptions of what evidence would be necessary or sufficient to demonstrate a practice as usual care. Descriptions included consistency with local practice or consistency across study sites, while others argued that local variation, national practice variation, and practices laid down in guidelines could all be determining factors as to what constituted usual care (Quotes 6.2–6.4).

Other dimensions of discussion included how usual care—as a description of practice—was differentiated from standard of care or practice standards defined as an expected level of care to be received. One example of this was concern about potential liability for adverse events when evaluating practices that differed from practice standards, such as those laid down by a Ministry or College. Others suggested that offering substandard care within a trial (either in the intervention or comparator arms) was unethical, even if it was consistent with usual care (Quotes 6.5 and 6.6).

Some interviewees further problematized the notion of usual care as a discrete and static comparator. For example, some suggested that in quickly developing specialties, or in low-resource settings, rapidly evolving practice would make it difficult to impose a single prior standard (even at individual study sites) as usual care (Quote 6.7). This latter point was especially emphasized in the context of reporting trial interventions. It was noted that when trials make claims to be applying interventions or comparators that represent usual care, this required clear reporting to understand what exactly constituted that care. For some, the reporting of interventions labelled as usual care in pragmatic RCTs was viewed as suboptimal (Quote 6.8).

A final aspect raised by interviewees was holding usual care as a standard when usual care was not necessarily based on evidence. Several interviewees argued that a rationale for pragmatic comparative effectiveness RCTs of existing practices was, in fact, that practices had developed based on expert opinion and not necessarily because of evidence of benefit. As such, explicitly examining those care standards was key.

Despite the definitional differences, variation in usual-care practice was important for consideration of equipoise. For example, several participants discussed how variation in usual practice regarding the use of treatment options would be indicative of uncertainty with respect to which was best and so would demonstrate community equipoise and justification for a trial.

## Discussion

In the present study we interviewed a range of stakeholders to explore their perspectives on ethical issues raised by pragmatic RCTs. The substantive areas of discussion were highly consistent with topics in the clinical trials literature, including risk [32–34], consent [35, 36], the governance of research activities [24, 37, 38], the selection of study participants [39], the roles and responsibilities of different stakeholders [40–42], and the publication and reporting transparency of trials [43–46], but also indicated that the available literature on ethics in pragmatic RCTs is relatively narrow in its focus. Our findings suggest that not only do we need to discuss familiar topics (such as appropriate consent approaches and participant protections) in new ways, but also that there are new questions (such as the different roles and responsibilities of a broader range of stakeholders in pragmatic RCTs) that need to be addressed.

These findings must be considered within the limitations of the study. First, interviews were conducted in English only. Consequently, issues pertaining to language or that may be specific to jurisdictions where English is not commonly spoken may be underrepresented. To try and mitigate this to a degree we interviewed respondents from a broad range of jurisdictions, including jurisdictions where the primary language is not English, and who had experience in a range of contexts. Second, patient partners and community members of study teams were recruited via the principal investigator of the identified study due to lack of a prior sampling frame. This may have introduced selection bias for a more positive outlook on pragmatic RCTs. Finally, participants were not provided a single definition of what constituted a pragmatic RCT. As such, definitions of pragmatic RCTs may have differed, yet the similarity of issues raised across participants suggests that any variation in definition had little influence on the key ethical issues they identified.

Central to much of the discussion was risk. This accords well with work by Kim and colleagues [34, 36], for example, who have not only questioned whether usual care interventions may be considered more than minimal risk, but also note the important role that risk assessment has for research ethics committees in their evaluation of the potential benefits and harms of research. While Chen and Kim [34] propose a framework for the analysis of risks, controversies on the nature of risks and the extent to which risks must be disclosed [47–51] illustrate that there is still disagreement on the relevant risks to be considered within trials, how these should be conceptualized, and how these are conveyed to trial participants. The development of guidance and best practices will not only need to engage with those stakeholders who review and oversee the conduct of pragmatic RCTs, such as research ethics review committees, but will necessitate discussions between researchers and the patient community with respect to understanding what are important risks for consideration and how these can be conveyed in an appropriate manner.

Despite the prominence of consent as a topic of discussion (e.g. [52–56]), an aspect raised here, but which is largely missing within the literature on pragmatic RCTs, was the need to engage with the broader legal norms that cover consent and the potential negative impact on public trust and the social licence for research [57] should alterations and waiver of consent be the subject of negative media coverage or legal challenges. This was identified by a 2014 report to the UK National Health Service, and which indicated that there may be important differences between types of trial with respect to additional consent needs and that guidance was required to balance legal requirements while minimizing burdens to the patient and the person seeking consent [58]. We suggest that such guidance remains necessary.

While there has been some discussion on the protections owed to “direct” and “indirect” participants of research [53] this debate has tended to focus on those proximally affected by a trial. The emphasis from interviewees in the present study was that there is a need for broader debate with respect to the individuals or groups affected by trials, and that this should also consider those more distal to the trial. A particular concern relating to trial participants was justice and equity. This emphasis on justice aligns well to the importance placed on including participants in pragmatic RCTs that reflect typical clinical populations [59], but also concerns expressed within previous studies of trial recruitment that found patients may be excluded by clinical researchers for practical reasons such as ability to travel or level of education rather than clinically relevant eligibility criteria [60]. Recent work by Johnson et al. indicates that the degree of pragmatism of an RCT may be perceived to change over time between design and implementation [61]. While such changes may reflect practical constraints or changes that need to be made, vigilance should be maintained with regard to equity considerations if changes affect the trial population. Moreover, the attention to equity conveyed here may also reflect a growing appreciation that justice requirements have not received sufficient attention within the research ethics literature, as emphasized by recent revisions to the Council for International Organizations of Medical Sciences (CIOMS) guidelines, which explicitly raise the topic of social justice in research [62–65].

The potential for different individuals or groups to be affected by a pragmatic RCT in different ways and to different extents raises the possibility that one should consider whether there are differing responsibilities to those affected by an RCT. Recent work has explored whether it would be ethically permissible to offer different financial payments to individuals enrolled within a trial if this addressed inequalities in the burdens imposed by trial participation [66] but, to date, the question of whether different obligations are owed to individuals differently affected by pragmatic RCTs has largely not been addressed.

While guidance, such as the Ottawa Statement on the Ethical Design and Conduct of Cluster Randomized Trials [67], identifies gatekeepers as serving an important role in cluster randomized trials, interviewees identified a broader set of individuals (such as healthcare administrators) who may feature in the development of pragmatic RCTs. How the roles and responsibilities of different stakeholders should be partitioned has received little attention in the literature on pragmatic RCTs. Consistent with the range of stakeholders identified in the present study, Faden et al. [40] identify healthcare administrators, payers, and purchasers as having important moral obligations in the context of “learning activities”, yet how these individuals should share responsibilities remains unclear. Whicher et al. [42] have proposed an ethical framework that outlines the roles of gatekeepers at different research stages, but this framework requires extension to specify to whom the stakeholders have responsibility within each of the identified research stages.

A final area of discussion was the concept of usual (or standard) care, a topic that has been a key point of discussion within recent trial controversies [47, 48, 50, 68]. As per Zwarenstein et al. [69], we do not wish to prescribe a specific definition of usual care. Rather we note that it is the responsibility of the trial team to determine and appropriately describe what usual care constitutes. However, consistent with the CONSORT extension for pragmatic trials [43] and the Template for intervention description an replication (TIDieR) guidelines [70], describing control interventions or co-interventions as “usual care” is not sufficient and they should be described in the same level of detail as the intervention arm. Notwithstanding the presence of these reporting guidelines, there is a paucity of research evaluating the extent to which the reporting of pragmatic RCTs meet recommendations.

## Conclusions

Based on this analysis we propose a set of questions that require further attention within the trial ethics literature (see Table 3). We believe that future work addressing these questions will contribute to ethically and empirically informed guidance and may greatly enhance the design and conduct of pragmatic RCTs.

**Table 3:** Key ethical questions for future evaluation

Key feature of pragmatic trials	Explanation	Ethical questions to be addressed
A key feature of pragmatic RCTs is that they often involve minimal modification of the clinical setting or organization and evaluate interventions commonly used in practice	Pragmatic trials may include elements of clinical practice, quality improvement, and research and these may have different implications for regulation or oversight	What are the ethically important distinctions between research, clinical practice and quality improvement in pragmatic trials? What criteria should be used to determine the type of oversight and regulation necessary for a pragmatic RCTs? How should these activities be regulated and on what basis would regulatory oversight differ?
Pragmatic RCTs seek to minimize the discrepancy between the trial and usual clinical care, which may include approaches to patient consent	Pragmatic RCTs may seek to recruit participants using altered consent approaches compared to standard written consent for research.	When are alterations and waivers of traditional informed consent appropriate in pragmatic RCTs? Does the general requirement for larger sample sizes and emphasis on external validity in pragmatic RCTs constitute a sufficient justification for waivers or alterations of consent (or may it raise concerns)?
	It can be difficult to determine which risks and benefits need to be disclosed to participants in the informed consent process when risks may be no more than usual care	What risks and benefits should be disclosed to participants in pragmatic RCTs of usual care interventions?
A key feature of pragmatic RCTs is that they seek to generate evidence applicable to a (the) wider population	There may be different actors (both within and outside of the trial in question) whose interests are affected by pragmatic RCTs in morally relevant ways	Who are the individuals or groups affected by the trial (and how do we determine who have legitimate claims on those conducting the trial)?
	Systemic barriers to trial participation may exist even when individuals are eligible	What are the responsibilities of identified stakeholders with respect to equity of access to pragmatic RCTs for those who are eligible? How should these responsibilities be determined?
A key feature of pragmatic RCTs is that they often include policy-relevant or health systems-relevant questions	There may be stakeholders who may influence the conduct or outcomes in pragmatic RCTs; they may have different responsibilities	Who are the stakeholders who have roles or responsibilities in relation to the trial (and how do we determine the individuals or groups who have roles)? What are their duties or responsibilities within the trial?
A key feature of pragmatic RCTs is that they may include a broader patient population with a broader range of risks/benefits and often assess interventions commonly used in practice (or already approved) that may be low risk	Due to inclusion of heterogeneous populations, pragmatic RCTs may include risks and benefits of differing magnitude which may accrue to different individuals or groups	How should we identify relevant risks and benefits to individuals or groups within a pragmatic RCT? How should these risks and benefits be evaluated?
	Pragmatic RCTs may not be exposing participants to treatments of unproven effectiveness; the use of existing treatments may have more well-characterized risk and benefit profiles^a^.	What criteria should be used to classify a pragmatic RCT as minimal risk?
External validity is key to pragmatic RCTs; in addition, they often include a comparator arm of “usual care” that is heterogenous	It can be difficult to determine what constitutes usual care and what meets legal definitions of accepted medical practice in pragmatic RCTs	What evidence is required to substantiate a claim that an intervention or comparator is usual care and if this would constitute minimal risk? Are two standardized arms within a usual care range actually usual care?
	Interpretation of a pragmatic RCT requires that the trial context (including the nature and variation within the intervention and comparator) is reported well	What are the essential elements of pragmatic RCTs that must be reported? How well are pragmatic RCTs reported, especially in relation to the constitution of usual care arms?

*RCT* randomized controlled trial

^a^This may not be the case for complex interventions, which may add further difficulty for risk assessment and evaluation for risk/benefit ratios

We suggest that, despite existing guidance on minimal risk and consent approaches [71], there is a need for further work to develop practically applicable frameworks for risk assessment in pragmatic RCTs and on the disclosure of risks to potential participants –especially where claims to routine, standard, or usual care are made. We propose that notions of usual care require greater conceptual exploration with respect to the identification of relevant risks and conduct of benefit-harm analyses, as well as practical guidance on how researchers and regulators should describe and interpret interventions or comparators described as usual care.

Furthermore, guidance with respect to when and how alternate consent approaches may be employed remains necessary. In particular, we suggest there is a need for greater discussion of the circumstances under which waivers of consent may appropriately be employed and the risks and benefits that need to be disclosed to participants, particularly in the context of pragmatic RCTs with a usual-care arm, which may change over time.

Finally, the roles and responsibilities of key stakeholders and the rights and protections owed to different populations who may be affected by a pragmatic RCT require further elaboration and guidance as to how these may differ from those in more explanatory RCTs. Here, special consideration should be given to justice and equity concerns, given the potentially heterogenous populations within pragmatic RCTs.

## Supplementary information

**Additional file 1.** PRECIS-2 Framework

**Additional file 2.** Trial expert interview guide.

**Additional file 3.** Patient partner and community member interview guide.

**Additional file 4.** List of investigators for the CIHR Ethics of Pragmatic Trials project.

## Data Availability

The datasets generated and/or analyzed during the current study are not publicly available due potential identifiability.

## Abbreviation

RCT: Randomized controlled trial
