# Supplementary material for: The ethical challenges raised in the design and conduct of pragmatic trials: an interview study with key stakeholders
Source: Trials. 2019 Dec 23;20:765. doi: 10.1186/s13063-019-3899-x (PMC6929346; doi:10.1186/s13063-019-3899-x)
Supplement: Supplementary file 1 — Additional file 1. PRECIS-2 Framework [file 13063_2019_3899_MOESM1_ESM.docx]

**Supplementary Table 1: PRECIS-2 domains and descriptors**

| PRECIS-2 domain | Description |
| --- | --- |
| Eligibility | To what extent are the participants in the trial similar to those who would receive this intervention if it was part of usual care? A more pragmatic trial would have criteria that ensure participants are essentially identical to those in usual care; a more explanatory approach would have lots of exclusions (e.g. those who don't comply, respond to treatment, or are not at high risk for primary outcome, are children or elderly), or uses many selection tests not used in usual care. |
| Recruitment | How much extra effort is made to recruit participants over and above what that would be used in the usual care setting to engage with patients? For example, a very pragmatic trial may have recruitment through usual appointments or clinic; a very explanatory trial may have targeted invitation letters, advertising in newspapers, radio plus incentives and other routes that would not be used in usual care. |
| Setting | How different is the setting of the trial and the usual care setting? For example, a very pragmatic trial may use identical settings to usual care; a very explanatory trial may include a single centre, or only specialised trial or academic centres. |
| Organisation | How different are the resources, provider expertise and the organisation of care delivery in the intervention arm of the trial and those available in usual care? For example, a very pragmatic trial may use identical organisation to usual care; a very explanatory trial may increase staff levels, give additional training, require more than usual experience or certification and increase resources. |
| Flexibility (delivery) | How different is the flexibility in how the intervention is delivered and the flexibility likely in usual care? For example, a very pragmatic trial may have identical flexibility to usual care allowing healthcare professionals to modify delivery of the intervention; a very explanatory trial may include a strict protocol, monitoring and measures to improve compliance, with specific advice on allowed co-interventions and complications |
| Flexibility (adherence) | How different is the flexibility in how participants must adhere to the intervention and the flexibility likely in usual care? For example, a very pragmatic trial may involve no more than usual encouragement to adhere to the intervention; a very explanatory approach may involve exclusion based on adherence, and measures to improve adherence if found wanting. |
| Follow-up | How different is the intensity of measurement and follow-up of participants in the trial and the likely follow-up in usual care? For example, a very pragmatic trial may have no follow up than would be the case in usual care; a very explanatory approach may have more frequent, longer visits, unscheduled visits triggered by primary outcome event or intervening event, and more extensive data collection. |
| Primary outcome | To what extent is the trial's primary outcome relevant to participants? For example, a very pragmatic trial would have an outcome is of obvious importance to participants; a very explanatory trial may use a surrogate, physiological outcome, central adjudication or use assessment expertise that is not available in usual care, or the outcome is measured at an earlier time than in usual care. |
| Primary analysis | To what extent are all data included in the analysis of the primary outcome? For example, a very pragmatic trial would use intention to treat with all available data; a very explanatory analysis may exclude ineligible post-randomisation participants, or include only completers or those following the treatment protocol |

Adapted from Loudon K, Treweek S, Sullivan F, Donnan P, Thorpe KE, Zwarenstein M. The PRECIS-2 tool: designing trials that are fit for purpose. *BMJ*. 2015;350:h2147 and https://www.precis-2.org/Help/Documentation/HowTo
