# Supplementary material for: The ethical challenges raised in the design and conduct of pragmatic trials: an interview study with key stakeholders
Source: Trials. 2019 Dec 23;20:765. doi: 10.1186/s13063-019-3899-x (PMC6929346; doi:10.1186/s13063-019-3899-x)
Supplement: Supplementary file 4 — Additional file 4. List of investigators for the CIHR Ethics of Pragmatic Trials project. [file 13063_2019_3899_MOESM4_ESM.docx]

**Supplementary Table 2: Ethics of Pragmatic Trials Team**

| **Name** | **Affiliation** |
| --- | --- |
| Adnan Ali (community partner) | Patient and Family Advisory Council. The Ottawa Hospital, Ottawa, Ontario, Canada |
| Jamie C. Brehaut | Ottawa Hospital Research Institute (OHRI), Ottawa, Ontario, Canada; School of Epidemiology and Public Health, University of Ottawa, Ottawa, Canada |
| Marion K. Campbell | Health Services Research Unit, University of Aberdeen, Health Sciences Building, Foresterhill, Aberdeen, UK, |
| Kelly Carroll | Ottawa Hospital Research Institute (OHRI), Ottawa, Ontario, Canada; |
| Sarah Edwards | Department of Science and Technology Studies, University College London, London, UK |
| Sandra Eldridge | Centre for Primary Care and Public Health, Queen Mary University of London, London, UK |
| Dean A. Fergusson | Ottawa Hospital Research Institute (OHRI), Ottawa, Ontario, Canada; Department of Medicine University of Ottawa, Ottawa, Canada; School of Epidemiology and Public Health, University of Ottawa, Ottawa, Canada |
| Christopher Forrest | Applied Clinical Research Center, Children’s Hospital of Philadelphia, Philadelphia, PA, USA |
| Bruno Giraudeau | Université de Tours, Université de Nantes, INSERM, SPHERE U1246, Tours, France; INSERM CIC1415, CHRU de Tours, Tours, France. |
| Cory E. Goldstein | Rotman Institute of Philosophy, Western University, London, Ontario, Canada |
| Ian D. Graham | Ottawa Hospital Research Institute (OHRI), Ottawa, Ontario, Canada; School of Epidemiology and Public Health, University of Ottawa, Ottawa, Canada |
| Jeremy M. Grimshaw | Ottawa Hospital Research Institute (OHRI), Ottawa, Ontario, Canada; Department of Medicine University of Ottawa, Ottawa, Canada; School of Epidemiology and Public Health, University of Ottawa, Ottawa, Canada |
| Karla Hemming | Institute of Applied Health Research, University of Birmingham, Birmingham, UK |
| Spencer P. Hey | Center for Bioethics, Harvard Medical School and Program on Regulation, Therapeutics, and Law at Brigham and Women’s Hospital, Boston MA. |
| Vipul Jairath | Division of Gastroenterology, Department of Medicine, Western University, London, Ontario, Canada; Division of Epidemiology and Biostatistics, Western University, University Hospital, London, Ontario, Canada |
| Terry P. Klassen | Children’s Hospital Research Institute of Manitoba, Winnipeg, Manitoba, Canada |
| Alex John London | Department of Philosophy and Center for Ethics and Policy, Carnegie Mellon University, Pittsburgh, PA, USA |
| Susan Marlin | Clinical Trials Ontario, Toronto, Ontario, Canada, |
| John C. Marshall | St. Michael’s Hospital, Department of Surgery, Toronto, Ontario, Canada |
| Lauralyn McIntyre | Ottawa Hospital Research Institute (OHRI), Ottawa, Ontario, Canada; Department of Medicine (Division of Critical Care), University of Ottawa, Ottawa, Ontario, Canada; School of Epidemiology and Public Health, University of Ottawa, Ottawa, Ontario, Canada |
| Joanne E. McKenzie | School of Public Health and Preventive Medicine, Monash University, Melbourne, Victoria, Australia |
| Trudy Mulder-Hall (community partner) | Hospice Palliative Care at Central West LHIN, Brampton, Ontario, Canada |
| Stuart G. Nicholls | Ottawa Hospital Research Institute (OHRI), Ottawa, Ontario, Canada; |
| P Alison Paprica | Institute of Health Policy, Management and Evaluation, University of Toronto, Toronto, Ontario, Canada, |
| Monica Taljaard | Ottawa Hospital Research Institute (OHRI), Ottawa, Ontario, Canada; School of Epidemiology and Public Health, University of Ottawa, Ottawa, Canada |
| Charles Weijer | Rotman Institute of Philosophy, Western University, London, Canada. |
| Merrick Zwarenstein | Centre for Studies in Family Medicine, Schulich School of Medicine and Dentistry, Western University, London, Ontario, Canada. |
